# Linguistic Pattern–Infused Dual-Channel Bidirectional Long Short-term Memory With Attention for Dengue Case Summary Generation From the Program for Monitoring Emerging Diseases–Mail Database: Algorithm Development Study

**DOI:** 10.2196/34583

**Published:** 2022-07-13

**Authors:** Yung-Chun Chang, Yu-Wen Chiu, Ting-Wu Chuang

**Affiliations:** 1 Graduate Institute of Data Science Taipei Medical University Taipei Taiwan; 2 Clinical Big Data Research Center Taipei Medical University Hospital Taipei Taiwan; 3 Department of Molecular Parasitology and Tropical Diseases School of Medicine, College of Medicine Taipei Medical University Taipei Taiwan

**Keywords:** ProMED-mail, natural language processing, dengue, dual channel, bidirectional long short-term memory

## Abstract

**Background:**

Globalization and environmental changes have intensified the emergence or re-emergence of infectious diseases worldwide, such as outbreaks of dengue fever in Southeast Asia. Collaboration on region-wide infectious disease surveillance systems is therefore critical but difficult to achieve because of the different transparency levels of health information systems in different countries. Although the Program for Monitoring Emerging Diseases (ProMED)–mail is the most comprehensive international expert–curated platform providing rich disease outbreak information on humans, animals, and plants, the unstructured text content of the reports makes analysis for further application difficult.

**Objective:**

To make monitoring the epidemic situation in Southeast Asia more efficient, this study aims to develop an automatic summary of the alert articles from ProMED-mail, a huge textual data source. In this paper, we proposed a text summarization method that uses natural language processing technology to automatically extract important sentences from alert articles in ProMED-mail emails to generate summaries. Using our method, we can quickly capture crucial information to help make important decisions regarding epidemic surveillance.

**Methods:**

Our data, which span a period from 1994 to 2019, come from the ProMED-mail website. We analyzed the collected data to establish a unique Taiwan dengue corpus that was validated with professionals’ annotations to achieve almost perfect agreement (Cohen κ=90%). To generate a ProMED-mail summary, we developed a dual-channel bidirectional long short-term memory with attention mechanism with infused latent syntactic features to identify key sentences from the alerting article.

**Results:**

Our method is superior to many well-known machine learning and neural network approaches in identifying important sentences, achieving a macroaverage F1 score of 93%. Moreover, it can successfully extract the relevant correct information on dengue fever from a ProMED-mail alerting article, which can help researchers or general users to quickly understand the essence of the alerting article at first glance. In addition to verifying the model, we also recruited 3 professional experts and 2 students from related fields to participate in a satisfaction survey on the generated summaries, and the results show that 84% (63/75) of the summaries received high satisfaction ratings.

**Conclusions:**

The proposed approach successfully fuses latent syntactic features into a deep neural network to analyze the syntactic, semantic, and contextual information in the text. It then exploits the derived information to identify crucial sentences in the ProMED-mail alerting article. The experiment results show that the proposed method is not only effective but also outperforms the compared methods. Our approach also demonstrates the potential for case summary generation from ProMED-mail alerting articles. In terms of practical application, when a new alerting article arrives, our method can quickly identify the relevant case information, which is the most critical part, to use as a reference or for further analysis.

## Introduction

### Background

Globalization and climate change have exacerbated the frequency and virulence of infectious diseases worldwide [1-3]. Climate and environmental changes play an undeniable role in changing disease ecology and transmission dynamics [4-6], with transboundary transmission also being frequently linked to international transportation [7]. Monitoring the region-wide or global infectious disease transmission patterns relies on intercountry collaborations to share disease surveillance information. The Program for Monitoring Emerging Diseases (ProMED)–mail [8] was launched by the International Society for Infectious Diseases in 1994 to collect global disease outbreak information on humans, animals, and plants [9,10]. Currently, ProMED-mail is the largest unofficial infectious diseases platform based on volunteer reporting, and it receives disease outbreak reports or research findings from different users (including individual scientists and governmental agencies) around the world. The EpiCore program, involving a worldwide network of public health professionals, was added in 2014 to scrutinize and verify the reported information [11].

Each report’s quality is enhanced through an expert-review process that includes reducing data redundancy and errors, which are common in social media reports. Reports from social media platforms such as Twitter and Facebook have been used to detect disease outbreaks in previous works, with Google Trends being a good example of web data providing early warning messages regarding influenza [12]. However, social media reports have at least three main limitations. The first involves unclear definitions. Many infectious diseases might share very similar clinical symptoms, making it difficult to differentiate them from simple keyword searches by users. Second, with the passage of time, the attention that people pay to disease outbreaks wanes. The third limitation relates to social media accessibility in different countries or groups of people: when researchers conduct long-term pattern analysis or multinational analysis of disease outbreaks, social media will introduce bias. These issues have been addressed by a few studies [13,14]. Social media and ProMED-mail might play different roles regarding dengue detection and analysis: whereas social media can be used to detect the emergence of dengue in the early stage, ProMED-mail can provide richer, more correct, and reliable epidemiological information, as well as continuous monitoring of that information [15-17].

Well-known contributions of ProMED-mail are the early reports of suspected cases of severe acute respiratory syndrome in China in 2002 and the Middle East respiratory syndrome coronavirus in Saudi Arabia in 2012 [11,18]. More recently, ProMED-mail data have been used to analyze a cholera outbreak in Africa, a vector-borne disease outbreak amid violent conflict in Syria, and global avian influenza outbreaks [19-21]. Thanks to >25 years of effort, huge amounts of disease outbreak information have been accumulated in the ProMED-mail database; however, the unstructured text format of the ProMED-mail report hampers the efficiency of scientific analysis. Most previous studies using the ProMED-mail database usually relied on labor-intensive review processes, making it difficult to analyze multiple diseases and broader study areas. However, natural language processing (NLP) can help because it is a powerful technique for extracting information from unstructured clinical or health records [22-24].

With the outbreak of COVID-19, sources of epidemic surveillance have received more and more attention, prompting the publication of several research papers. Nonetheless, few studies have taken advantage of NLP technology for the development or analysis of data from ProMED websites. Carrion and Madoff [11] have noted that every season, ProMED would publish a word cloud of epidemics in various regions to show epidemics that have been particularly severe in each region in the current season. Taking 2016 as an example, alerting reports from all over the world were processed by NLP technology to produce a visualized word cloud in which dengue is the keyword for entire Southeast Asia. In addition, Kim et al [25] developed a deep learning approach to automatically recognize the relevant information that is necessary to deal with potential disease outbreaks; this is consistent with our view. Their study used 2 approaches—convolutional neural network (CNN) and bidirectional long short-term memory (BiLSTM) [26,27]—to classify the sources of the texts about infectious diseases in the alerting articles, and they achieved an overall accuracy rate of 92.9%.

In recent years, factors such as climate, weather, and culture have stimulated global epidemic monitoring, from which it has become clear that dengue fever in Southeast Asia still poses a serious threat [28,29]. Dengue incidence has increased significantly around the world in the last 2 decades [30], with an estimated 390 million infections per year, of which 96 million exhibit clinical symptoms. Each year, approximately 3.9 billion people are at risk of infection with dengue viruses, with 2 million severe cases and 2100 deaths [31]. Although dengue infection is prevalent in 129 countries, 70% of all cases are located in Asia. The reported number of dengue cases has increased >8-fold over the last 2 decades, and most deaths have occurred in younger age groups. As there is currently no effective vaccine or treatment available for dengue infection, dengue surveillance information is imperative for disease control and prevention.

For these reasons, our research focuses on Southeast Asia. As the alerting articles from ProMED-mail are lengthy (an average of 1872 words and 82 sentences in Asian-related dengue fever alerting articles), it is important to develop a summary generation system to assist relevant researchers to become more proficient in monitoring the pandemic. In general, case information is related to the outbreak location, time, and patient [32]. The combination of these 3 types of information constitutes the coincidence of occurrence of case information. However, although NLP research for information extraction has flourished [33-36], it is not easy to determine whether all the important case information is contained in a single ProMED-mail alerting article. For example, as digits usually represent the number of cases, the appearance of important case information is often accompanied by the appearance of numbers. However, in the sentence “CDC deputy director-general Chuang Jen-Hsiang said on Wednesday [August 7, 2019] that the patient also has underlying diseases, which is why he was only diagnosed with dengue after 2 screenings and multiple hospital visits,” the digit “2” represents frequency, rather than the number of cases. It thus cannot become one of the sentences in the summary, although it contains dengue-related keywords such as disease, dengue, and diagnose. Moreover, location and time are also relevant information for important cases. We thus assume intuitively that if the location and time are mentioned in the same sentence, it may more likely describe relevant information about the case. However, although the sentence “Since 18 Nov [2006], Kaohsiung County City health authorities reinforced implementation of the mosquito-elimination campaign” mentions location and time, the content obviously does not contain information related to any infection outbreak.

### Objectives

The specific aim of this research was therefore to extract abstract sentences that contain important epidemiological information on dengue incidence in Southeast Asia. Our proposed method can enhance the decision-making efficiency of epidemic monitoring units by quickly and automatically generating summaries of alerting articles. This is particularly important during the current COVID-19 pandemic. Specifically, our method first decomposes an alerting article from ProMED-mail into sentences. Next, the sentences are classified into 2 categories in accordance with their syntactic characteristics. Finally, the proposed deep neural network–based method integrates linguistic patterns and latent syntactic features to identify important sentences as the basic unit of summary generation. The experiment results based on real-world data sets demonstrate that the proposed method successfully exploits the syntactic, semantic, and contextual information relevant to epidemiological information on dengue. Consequently, our method not only outperforms many well-known information extraction methods but also achieves a satisfaction rating of 84% for the abstractive sentence summarization of dengue alerting article data.

## Methods

### Data Corpus

The aim of this study was to develop a method to extract sentences that convey dengue case information in ProMED-mail alerting articles and then automatically generate summaries. However, to the best of our knowledge, there is no official data set for crucial sentences extraction with regard to dengue. For this reason, we compiled our own data corpus for method development and performance evaluation. To do this, we first collected all the articles from 1994 to 2019 on the ProMED-mail website as a preliminary corpus. Next, we used the country name to further extract Southeast Asia articles and then verified that the title contained “Dengue/DHF (dengue hemorrhagic fever) updates” to indicate a series of dengue fever alerting articles. To facilitate the efficiency of the corpus construction, we sampled 15% of the instances from the data set for annotation for a total of 129 articles that contained 965 sentences. Next, the data set was annotated by 2 experts who are medical professionals with high English proficiency. Before the annotation stage, we conducted a training to ensure that the annotators had a common understanding of what defines crucial sentences in a dengue summary. We provided each annotator with 20 instances of both positive and negative cases during the training stage. A third expert acted as an arbiter for verifying the annotations. During the actual labeling process, the annotators labeled 246 instances as crucial sentences (Cohen κ=0.895, which indicates that the interobserver agreement of our annotated data corpus is reliable [37-39]). The final annotation results were used for the performance evaluation of the proposed model. It is worth noting that 80.1% (197/246) of the crucial sentences were composed of multiple clauses with complex syntactic structures (Table 1). Furthermore, 34.6% (85/246) of the sentences contained digits that did not convey case information (eg, “Dengue virus circulating type 1” and “20-34 mosquitoes or mosquito larvae found in every 100 households”). In light of these sentence statistics, it is clearly a challenging task to extract sentences that convey case information. The corpus has been released to promote further research and is available at the Taipei Medical University Dengue Case Corpus [40].

**Table 1:** The statistics of our corpus (N=965)^a^.

	Number of sentences, n (%)	
	Single-clausal sentences	Multiclausal sentences
Dengue case sentences (n=246)	49 (19.9)	197 (80.1)
Non-dengue case sentences (n=719)	407 (56.6)	312 (43.4)

^a^Number of paragraphs: 129, number of sentences: 965, number of single-clausal sentences: 456, and number of multiclausal sentences: 509.

**Figure 1:**
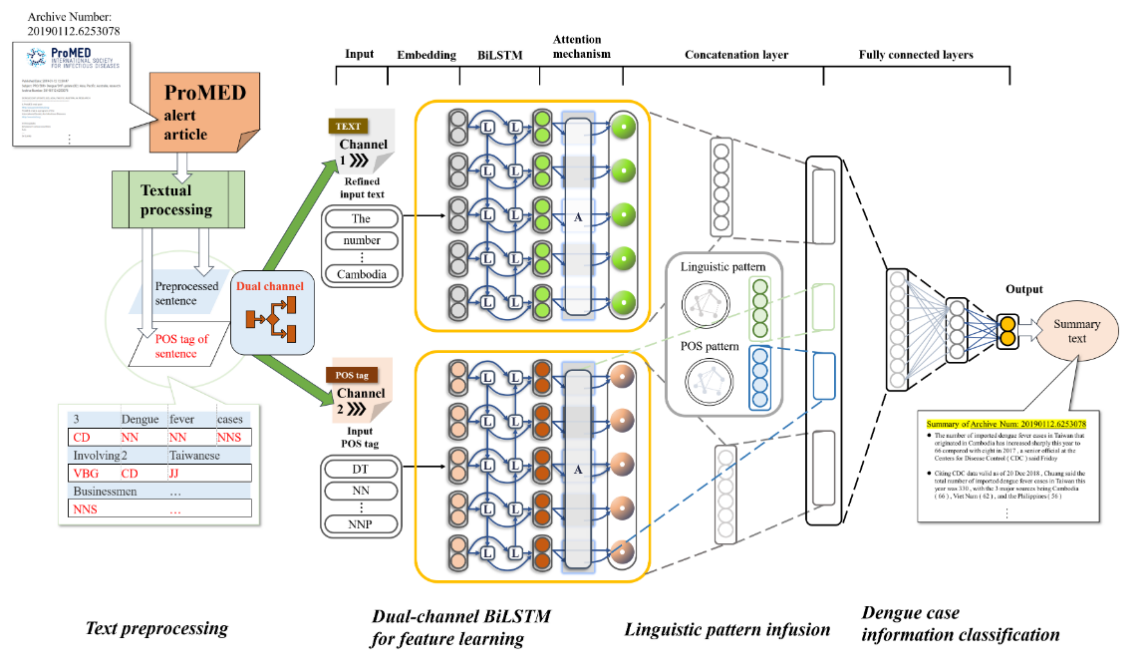
Overview of the proposed framework. A: attention layer; BiLSTM: bidirectional long short-term memory; CD: cardinal number; DT: determiner; JJ: adjective; L: forward long short-term memory layer and backward long short-term memory layer; NN: noun, singular or mass; NNS: noun, plural; POS: parts of speech; ProMED: Program for Monitoring Emerging Diseases; VBG: verb, gerund, or present participle.

Figure 1 presents the overview of the proposed framework, which automatically detects summary sentences that contain case-related information in a set of documents of the dengue alerting series written in English. In this research, we used a linguistic pattern–infused BiLSTM with an attention mechanism neural network for dengue case information extraction. That is, to extract sentences that convey dengue case information from alerting articles from ProMED-mail, we treated dengue case information extraction as a binary classification problem that can be formulated as follows. Let *W* = {*w_1_*,…, *w_k_*} be a set of words and *S* = {*s_1_*,…, *s_m_*} be a set of sentences from a set of alerting articles. Each sentence *s* comprises a set of words such that *sW*. The goal of this task was to decide whether a sentence *s_j_* expresses dengue case information. Our framework consisted of 4 main procedures: text preprocessing, dual-channel BiLSTM (DuBiLSTM), linguistic pattern infusion, and dengue case information classification. Further details of each procedure are provided in the following sections.

### Text Preprocessing

Preprocessing is a critical task that needs to be performed before feeding data into a neural network. When a report document *d_n_* is entered, we first decompose it into a set of paragraphs *P* = {*p*_1_,..., *p*_i_} and obtain the sentence collections *S* = {*s_1_*,..., *s_j_*} through sentence segmentation for each paragraph. Next, we break a sentence into tokens and tag the parts of speech (POS) *T* = {*t*_1_,..., *t*_k_} using the Natural Language Toolkit package [41]. As dengue case information can be narrated in a sequence of clauses, we recognize 2 types of sentences, namely single-clausal sentences and multiclausal sentences. Moreover, as frequently used words are generally not helpful for identifying dengue case information, we removed the stop words as well as punctuation marks (commas and semicolons) in the sentences.

### DuBiLSTM With Attention Mechanism

In this research, we developed a DuBiLSTM with an attention mechanism neural network to learn latent semantic features behind both alert article texts and shallow paring information. The embedding layer is first used to transform the input tokens and POS tags of a sentence into 300D vectors. We used Global Vectors for Word Representation pretrained word embeddings (ie, glove.6B) to transform sentences of the alerting articles into 300D vectors. The POS embeddings are learned from the embedding layer of our model with continuous bag-of-words mode. Specifically, a sentence is represented by *s* = {*w_1_*,..., *w_k_*}{*pos_1_*,..., *pos_k_*}, its corresponding word vector is *v_w_* = {*v_w_1_*,..., *v_w_n_*}, and its POS vector is *v_pos_* = {*v_pos_1_*,..., *v_pos_n_*}, which are the inputs of the model.

Compared with the original recurrent neural network, the reason for the improvement of long short-term memory (LSTM) is its special design. LSTM defines and maintains an internal memory cell state throughout the life cycle to establish temporal connections. This internal memory cell state is the most important element of LSTM’s structure. The LSTM model consists of a series of identical timing modules. In addition to the original input, LSTM has 3 designs—forget gate, input gate, and output gate—that determine whether the input is important enough to be remembered and whether it can be output.

The details are described herein. Suppose there are 3 element-wise functions that help to calculate the next moment by the previous moment and this moment where σ(.) is a sigmoid function, tanh(.) is a hyperbolic tangent function, and 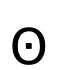 is the product. We also have x*_t_* ∈ 
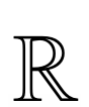
*^d^* and h*_t_* ∈ 
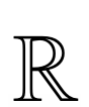
*^h^* denoting the input vector and the hidden state vector at moment *t*, respectively, whereas *U* ∈ 
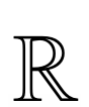
*^h^*^×^*^h^* and *W* ∈ 
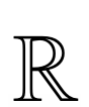
*^h^*^×^*^d^* indicate the weight metrices of gates or cells for input vector x*_t_* and hidden state vector h*_t_*, respectively, and *b* ∈ 
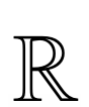
*^h^* indicates the weight metrices of gates or cells for the bias vector, where the superscripts *d* and *h* refer to the number of input features and number of hidden units, respectively. The forget gate at the moment *t* f*_t_* ∈ 
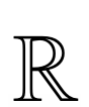
*^h^* determines the information to be forgotten by outputting a number in (0, 1), in line with the following equation:

f*_t_* = σ(*W_f_*h*_t_*_–1_ + *U_f_*x*_t_* + *b_f_*) **(1)**

With regard to the second mechanism, the input gate of LSTM then decides what new information input should be kept by calculating i*_t_* ∈ 
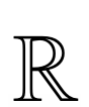
*^h^* and c ~*_t_* ∈ 
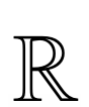
*^h^* and combining the 2 parameters in the light of the following equations:

i*_t_* = σ(*W_i_*h*_t_*_–1_ + *U_i_*x*_t_* + *b_i_*) **(2)**

c ~*_t_* = tanh(*W_c_*h*_t_*_–1_ + *U_c_*x*_t_* + *b_c_*) **(3)**

c*_t_* = f*_t_*

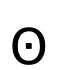
 c*_t_*_–1_ + i*_t_*

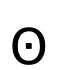
 c ~*_t_*
** (4)**

The third special mechanism is the output gate. This represents which parts of the cell state should be outputted based on the following equations, where h*_t_* ∈ 
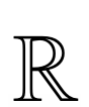
*^h^* represents the hidden state vector, also known as the output vector:

o*_t_* = σ(*W_o_*h*_t_*_–1_ + *U_o_*x*_t_* + *b_o_*) **(5)**

h*_t_* = o*_t_*

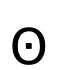
 tanh(c*_t_*) **(6)**

However, the information in the LSTM network is a 1-way transmission, and LSTM can only use past information, not future information. BiLSTM can consider both past and future data information by connecting 2 LSTM networks with opposite timings in the same output. The forward andbackward LSTMs can obtain, respectively, the past and future data information of the input sequence. The hidden state *H_t_* of BiLSTM at time *t* includes forward 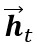 and backward 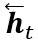:



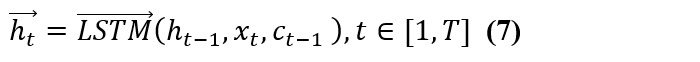





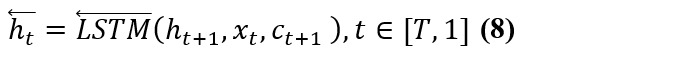





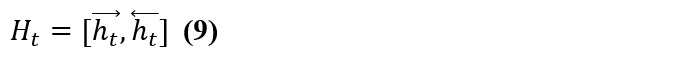



In addition, as the attention mechanism can allocate more attention to important information and less to other information, the receiving sensitivity and processing speed of information in the focused attention area are greatly improved. The attention function can softly map the combination of query *Q* and a set of key-value pairs {*K,V*} to some notable outputting results, where *Q* = {*Q*_1_,..., *Q_N_*} and {*K*,*V*} = {(*K*_1_,*V*_1_),..., (*K*_M_,*V*_M_)}. Furthermore, the multihead attention mechanism would convert *Q*, *K*, and *V* into *H* subspaces in the first step, with various and learnable linear projections, as the following transforming equation shows:



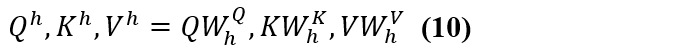



where {*Q^h^*, *K^h^*, *V^h^*} are the input query, key, and value of the *n*th head, respectively; 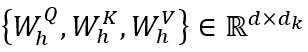 represents the parameter matrices at the same time; and *d* and *d_k_* indicate, respectively, the dimension of the model and its subspace. In the second step, *H* attention functions are implemented in parallel to generate the output states *O* = {*O*^1^,..., *O^H^*}, where any *O^h^* in *O* is defined in the following equation:



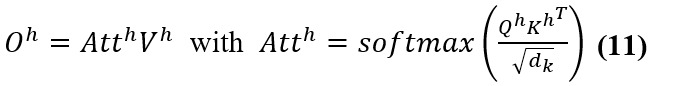



where *Att^h^* is the attention distribution that comes from the *h*th attention head. Finally, the output states *O* are concatenated with each other and then connected with distinctively generated features for the next stage.

### Linguistic Pattern Infusion

The human perception of a dengue case information alert involves identifying a relevant lexicon or semantic content to rapidly narrow down the scope of possible candidates. For instance, when an expression contains strongly correlated words such as “dengue” and “total” at the same time, it is natural to conclude that this is probably an expression about a dengue case. These lexical indicators can help explain how humans can skim through an article to quickly capture the dengue case information expressions. Therefore, we used log-likelihood ratio (LLR); it is an effective feature selection method that can generate representative patterns from sequences of dengue case information expressions [42-44]:



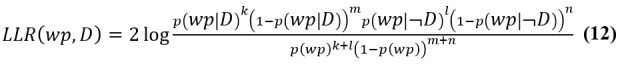



Given a training data set composed of binary labels for representing sentences that describe dengue case information (*D*) or not (*¬D*), we pair words from sentences to generate a set of co-occurring word pairs WP = {*wp_1_*,..., *wp_f_*} and POS pairs PP = {*pp_1_*,..., *pp_g_* }. The LLR uses the following mechanism to calculate the likelihood that the occurrence of a word pair and a POS pair in the dengue case information is not random. To illustrate, we take the LLR calculation for a word pair, where *N*(*D*) and *N*(*¬D*) are the numbers of positive and negative sentences, respectively. *N*(*wp*^D), which is denoted as *k*, is the number of alert articles containing *wp* and *D* simultaneously, whereas *N*(*wp*^*¬D*), which is denoted as *l*, is the number of negative sentences that include *wp*. To further simplify the formula, we also define *m* = *N*(*D*) – *k* as the number of sentences containing *D* without the word pair *wp* and n = *N*(*¬D*) – *l*, which means the number of sentences with neither *D* nor *wp*. A maximum likelihood estimation is conducted to obtain probabilities *p*(*wp*), *p*(*w*|*D*), and *p*(*w*|^*D*) by calculating the log-likelihood of the hypothesis that the presence of *wp* in set *D* is not random. A word pair with a large LLR value is therefore closely associated with the expression of dengue case information. We rank all the word pairs by every LLR value in the training data, and the top 50 word pairs that describe dengue case information and those that do not are selected as linguistic patterns for positive and negative sentences, respectively. The same procedure is adopted to calculate the LLR value for the POS pairs for the compilation of POS patterns.

Next, we integrate the generated linguistic patterns and POS patterns into a DuBiLSTM with an attention mechanism by concatenating both positive and negative vectors (which are composed of 60 and 25 dimensions, respectively) with the LLR value of the matched patterns (ie, 170D pattern vectors). As the LLR values of linguistic patterns and POS patterns indicate the weight associated with positive and negative sentences, merging the linguistic pattern and POS pattern features into a DuBiLSTM with an attention mechanism is discriminative.

Finally, the direct splicing strategy is used to fuse pattern features with latent semantic features from a DuBiLSTM with an attention mechanism neural network. The calculation formula for this is as follows:

*F* = *PF*⊕*LSF*
** (13)**

where PF=pattern features and LSF=latent semantic features.

### Dengue Case Information Classifier

The final step in our framework involves constructing a classifier to predict the labels through the 3 fully connected layers and the activation layer and then to output the distribution probability of the labels. In the fully connected layer, the model maps the fused feature vector to the instance label space. In the output layer, the softmax function is used for normalization, and the output of the fully connected layer is converted into the approximate probability value *y* for each category. The calculation formula is as follows:



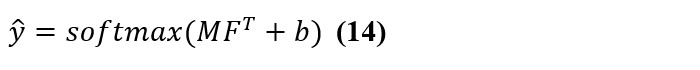



where *M* is the parameter matrix of the connection layer, *F* is the characterization of the fusion-distributed characteristics, *b* is the bias, and softmax is a normalization function. Although the 3 fully connected layers increase the computational cost, the classifier efficiently learns weights through the neuron layer [45-47]. The neurons in each layer will be connected to the neurons in the next layer. Considering the convergence rate, the rectified linear unit (ReLU) function is used as an activation function for nonlinear operation. This can easily cause overfitting in model learning; therefore, to avoid this, we use the dropout mechanism to correct for overfitting [48]. The 2 probabilities are predicted for negative and positive, and the larger probability will be taken out to become the final prediction result through the softmax function.

In addition, the Adam optimizer [49] was chosen to optimize the loss function of the network. The model parameters are fine-tuned by the Adam optimizer, which has been shown to be an effective and efficient backpropagation algorithm. We use the cross-entropy function as the loss function because it can reduce the risk of a gradient disappearance during the process of stochastic gradient descent; this is why it often performs better than the classification error rate or the mean square error [50]. The loss rate of the model can be calculated using the following equation:



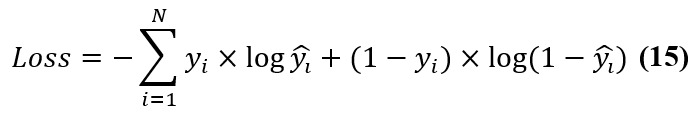



where *N* is the number of training samples, *y* is the label of the sample, and 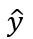 is the output of the model.

### Comparative Analysis Models

To conduct a comprehensive evaluation of the proposed method, we also developed baselines of the machine learning model and deep neural networks to estimate the significance of our approach and the performance variation in different classification systems. Our first baseline uses a tokenized representation evaluated on the radial basis function kernel–based support vector machine (SVM) [51]. This system learns the statistical relevance of each token in a clinical record within different syntactic and semantic contexts. Next, 2 ensemble learning approaches were also implemented for comparison. The first is random forest (RF), an ensemble learning method for classification that constructs a multitude of decision trees (DTs) adopting term frequency–inverse document frequency text representation. The other model is the classifier extreme gradient boosting (XGB), which is a gradient boosting DT that integrates multiple learners for classification problems [52]. We included XGB in this study because it has been validated on real-life large-scale imbalanced data sets and solves many data science–related problems in a fast and accurate way [53]. Finally, the 3 deep neural networks were compared for performance evaluation. The first deep learning model is a class of feedforward artificial neural networks called multilayer perceptron (MLP) [54]. We constructed an MLP through 3 fully connected dense layers afterward the input layer (the model was constructed by adding 3 hidden layers between the input layer and the output layer, where hidden layers are responsible for feature extraction to help output layer to do classification work). In addition, we also adopted a whole-text multikernel CNN model using static word embeddings [55,56] of instances (CNN for text) as another baseline. The last deep learning model that we included in the comparative analysis is the LSTM recurrent neural network, which is capable of learning order dependence in a text sequence and is widely used in NLP research.

To examine the incremental performance that benefits from the proposed method, the BiLSTM and DuBiLSTM are also listed as comparators. To serve as a basis for comparison, we also included naïve Bayes [51,57-59] and DT [51] as baselines.

## Results

### Evaluation Metrics

In our experiments, the performance evaluation metrics included precision, recall, and F1 score. In general, there is a trade-off between precision and recall. As the 2 metrics evaluate system performance from different perspectives, a single metric that balances (ie, averages) the trade-off is essential. The F1 score is the harmonic mean of precision and recall, and as it is generally close to the minimum of the 2 values, it can be considered an attempt to find the best possible compromise (balance) between precision and recall [51]. The F1 score is also deemed a conservative metric that prevents the possible overestimation of system performance because the harmonic mean is always less than, or equal to, the arithmetic mean and geometric mean. For this reason, the F1 score is extensively used to judge the superiority of information systems [51]. We used the macroaverage to compute the average performance, and to obtain reliable verification results, we adopted a 10-fold cross-validation approach [60]. Our model was implemented with Keras [61] under the following configurations: the dropout probability was set at 0.35 after each layer, loss function was categorized as cross-entropy, ReLU activation was applied to the dense layer, and training was set at 40 epochs.

### Model Comparisons

The model performance is shown in Table 2. First, the naïve Bayes classifier, which is a conditional probability–based approach using bag-of-words feature space with term frequency–inverse document frequency term weighting, only achieved a mediocre performance. As this classifier only considers surface word weightings, it has difficulty representing interword relations, and its overall F1 score is only 70.34%. By contrast, the DT further learns keyword weighting for representation through an entropy-based feature selection method. Hence, the DT is able to obtain significant improvement. The XGB obtained better performances because it is able to integrate multiple machine learning algorithms using the gradient boosting mechanism to optimize the loss function. Likewise, the RF integrates multiple DTs through ensemble learning to optimize prediction results. Therefore, the overall performance of the XGB and RF are similar, with both achieving F1 scores of approximately 85%. It is worth mentioning that the prediction performance of an SVM achieves an F1 score of approximately 90%. This is because the SVM can solve nonlinear obstacles and build models based on learned word and phrase correlations in context, thereby enhancing classification performance. When comparing the deep learning approaches, the performance of an MLP is similar to the ensemble learning–based approaches. The redundancy and inefficiency might be caused by the large number of parameters in the fully connected neural network structure. The learned weighting is therefore unreliable and leads to the lowest performance among all neural models considered in this study. In contrast to the MLP, CNN for text and LSTM have better performances that achieve F1 scores of approximately 90%. This indicates that both deep neural network models efficiently represent textual information and learn the context of the ProMED alerting articles to identify dengue case information. It is worth noting that our method can extract latent linguistic features from ProMED alerting reports because learned word and POS embeddings are adopted to represent syntactic and context relations. Moreover, we used discriminative patterns to encode the characteristics of collocation relationships to capture descriptors of dengue within the alert reports. Consequently, our method achieves the best overall precision, recall, and F1 score among the compared methods.

**Table 2:** The performance results of the compared methods.

System	Negative, precision; recall; F1 score (%)	Positive, precision; recall; F1 score (%)	Macroaverage, precision; recall; F1 score (%)	*P* value
NB^a^	81.69; 99.30; 89.64	94.51; 34.96; 51.04	88.10; 67.13; 70.34^b^	<.001
DT^c^	95.79; 85.40; 90.29	67.59; 89.02; 76.84	81.69; 87.21; 83.57^b^	<.001
RF^d^	96.53; 88.87; 92.54	73.60; 90.65; 81.24	85.06; 89.76; 86.89^b^	<.001
SVM^e^	94.12; 95.69; 94.90	86.75; 82.52; 84.58	90.43; 89.10; 89.74^b^	<.001
XGB^f^	92.55; 91.52; 92.03	75.98; 78.46; 77.20	84.26; 84.99; 84.61^b^	<.001
MLP^g^	94.64; 90.82; 92.69	76.00; 84.96; 80.23	85.32; 87.89; 86.46^b^	<.001
CNN^h^ for text	94.47; 94.99; 94.73	85.12; 83.74; 84.43	89.80; 89.37; 89.58^b^	<.001
LSTM^i^	94.72; 94.85; 94.79	84.90; 84.55; 84.73	89.81; 89.70; 89.76^b^	<.001
BiLSTM^j^	95.74; 93.88; 94.80	83.08; 87.80; 85.38	89.41; 90.84; 90.09^k^	.94
DuBiLSTM^l^	95.89; 94.16; 95.02	83.78; 88.21; 85.94	89.84; 91.18; 90.48^k^	.95
Our method	97.72; 95.27; 96.48	87.12; 93.50; 90.20	92.42; 94.38; 93.34	—^m^

^a^NB: naïve Bayes.

^b^*P*<.001 (a chi-square test was applied to determine whether our method signiﬁcantly improves performance in comparison with other methods).

^c^DT: decision tree.

^d^RF: random forest.

^e^SVM: support vector machine.

^f^XGB: extreme gradient boosting.

^g^MLP: multilayer perceptron.

^h^CNN: convolutional neural network.

^i^LSTM: long short-term memory.

^j^BiLSTM: bidirectional long short-term memory.

^k^*P*>.05 (a chi-square test was applied to determine whether our method signiﬁcantly improves performance in comparison with other methods).

^l^DuBiLSTM: dual-channel bidirectional long short-term memory.

^m^Not available.

**Figure 2:**
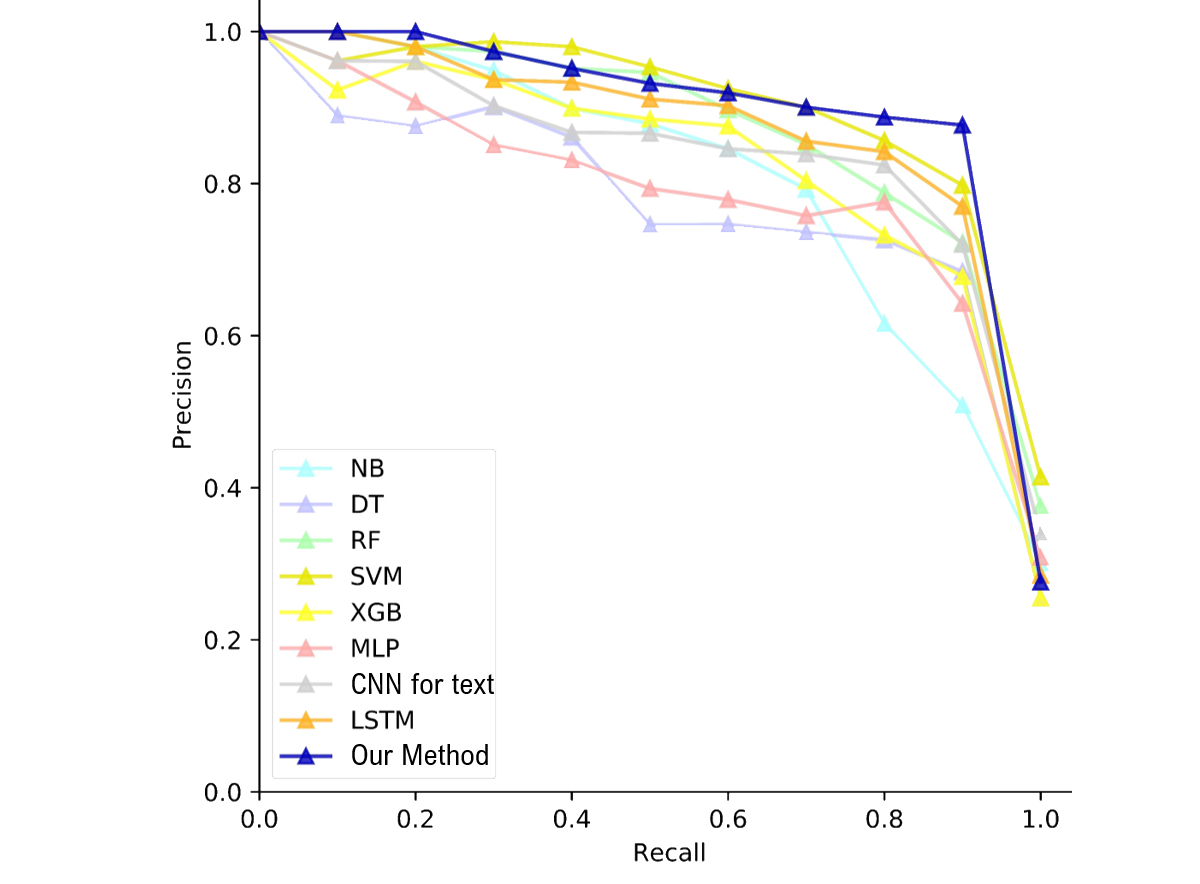
The precision recall curves of the compared methods. CNN: convolutional neural network; DT: decision tree; LSTM: long short-term memory; MLP: multilayer perceptron; NB: naïve Bayes; RF: random forest; SVM: support vector machine; XGB: extreme gradient boosting.

Finally, we evaluated the performances of the compared methods using 11-point precision recall curves [62]. To plot these curves, the evaluated sentences were sorted according to their prediction scores. Figure 2 shows that the precision scores of our method at the 11 recall levels are superior to those of the compared methods. In other words, our method is able to most accurately extract sentences that convey dengue case information.

To summarize, the DuBiLSTM is able to learn the latent syntactic and semantic information of a text, and the attention mechanism can further highlight the important elements in context. The linguistic patterns are successfully integrated into the neural network to capture discriminative collocation of latent features. Consequently, our method significantly outperforms the compared methods and achieves a remarkable dengue case information extraction performance.

## Discussion

### Principal Findings

This study describes a new method for identifying dengue case information by using BiLSTM with an attention mechanism enriched with linguistic patterns. As the results show, BiLSTM can consider contextual information more efficiently. Through its bidirectional mechanism, the output for any current moment is not only related to a previous state but may also be related to a future state. The DuBiLSTM can yield an even more slightly improved overall performance because of the benefits accruing from the enhancement of the precision and recall of both positive and negative categories. This indicates that the dual-channel framework is able to generate more shallow linguistic features for BiLSTM. It is noteworthy that our method achieves the best performance. As the generated linguistic pattern can examine the content of sentences to identify dengue case information, it does not conflict with the DuBiLSTM, which analyzes syntactic and semantic information in the sentences.

As a consequence, combining BiLSTM and DuBiLSTM improves the system performance and achieves a remarkable performance on the Taipei Medical University Dengue Case Corpus. The high proportion of dengue case information expressions can be identified by the generated linguistic patterns. For instance, the positive sentence “Taiwan recorded another 7 cases of dengue fever [Wednesday, September 5, 2018], bringing the total number so far this year to 81 and prompting stronger calls by the relevant authorities for greater public cooperation to prevent the spread of the mosquito-borne disease in the peak season*.*” is correctly detected as dengue case information sentence through the successful match of the generated pattern *[total]-[dengue]* and *[reported]-[dengue]*. It shows that identifying sentences with matched patterns can enhance the performance to discriminate the dengue case information extraction.

**Figure 3:**
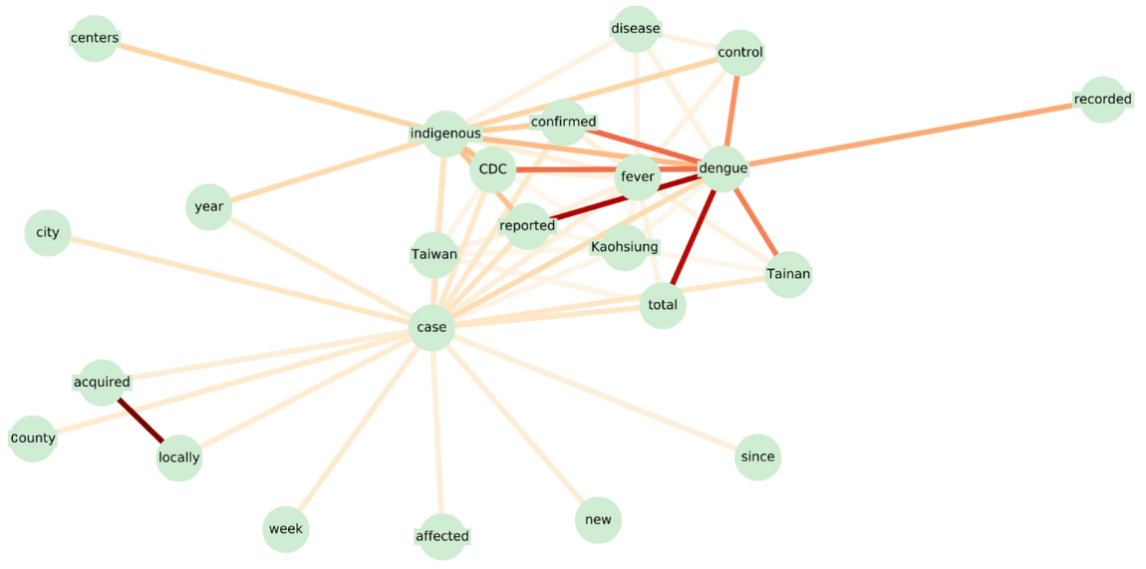
The network visualization for generated linguistic patterns. CDC: Taiwan Centers for Disease Control.

**Figure 4:**
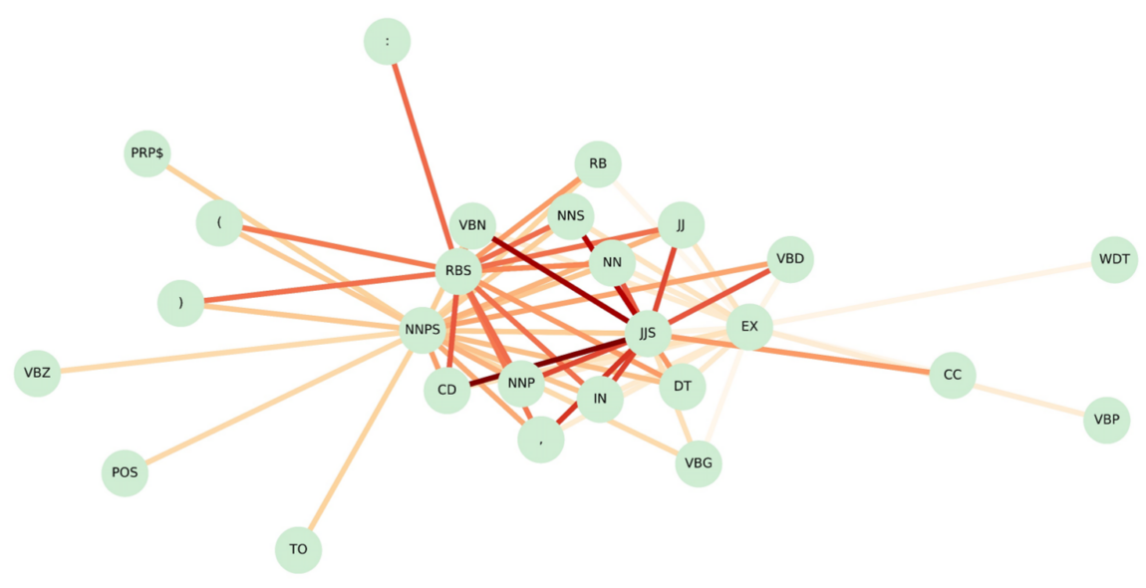
The network visualization for generated parts-of-speech (POS) patterns. CC: coordinating conjunction; CD: cardinal number; DT: determiner; EX: existential there; IN: preposition or subordinating conjunction; JJ: adjective; JJS: adjective, superlative; NN: noun, singular or mass; NNP: proper noun, singular; NNPS: proper noun, plural; NNS: noun, plural; PRP$: possessive pronoun; RB: adverb; RBS: adverb, superlative; TO: to; VBD: verb, past tense; VBG: verb, gerund, or present participle; VBN: verb, past participle; VBP: verb, nonthird person singular present; VBZ: verb, third person singular present; WDT: wh-determiner.

As shown in Figures 3 and 4, we visualized the collocation of words and POS for further observation, where nodes and edges represent the linguistic pattern, with the depth of the edge denoting the weight value (ie, LLR) of the collocation. We can observe the appearance of linguistic patterns in Figure 3, such as *[dengue]-[reported]* and *[locally]-[acquired]*, indicating that the sentence is more likely to be crucial. In addition, we also noticed that the word *case* has radial edges, which suggests that many discriminative linguistic patterns are composed of the word *case*. This is because case-related information often mentions the term *case*. For instance, “Dengue [reported] 100 cases locally acquired; Municipality most affected: Kaohsiung City,” which is a very typical example containing accurate case information. In addition, from Figure 4, we observe from the POS pattern network that the more important POS collocations are *[JJS]-[CD]*, where *JJS* stands for *adjective, superlative* and *CD* stands for *cardinal number*, and *[JJS]-[NN]*, where *NN* stands for *noun, singular or mass* (the detailed meanings of POS tags are provided in the URL [63]. This is because the case numbers mostly occur in digit form, and in this situation, the POS tag belongs to *CD*, which is then combined with adjectives and nouns to complete the description of the case information.

Table 3 lists the errors in the sentences of single-clausal and multiclausal types. As shown in the table, the total error rate of the proposed method is 6% (58/965). The individual error rates of single-clausal and multiclausal sentence types are 3.1% (14/456) and 8.6% (44/509), respectively. This indicates that dengue case information in multiclausal sentences is difficult to detect. This is because the syntactic structures of multiclausal sentences are so complex that they confuse the pattern-matching process. As a result, the matched linguistic and POS patterns are prone to errors that affect the correctness of pattern representation and the performance of the corresponding detection. We also observed from the results that a vast proportion of false positives, that is, negative instances incorrectly identified as positive, occurred because the sentences expressed global dengue case information instead of expressing information from the observed country of the alert. For example, our model incorrectly classified “This alarming, particularly considering 96 million cases of symptomatic dengue year worldwide*.*” as a positive sentence. However, the sentence conveys the aggregation of an annual and global pandemic situation, rather than representing a piece of detailed case information, although it contains the highly associated words *case* and *dengue*. For the false negatives, we noticed that a sentence may be split into several fragments because of the writing style. This can result in an incomplete context and thus unclear semantics behind the text. For instance, the sentence “Locality affected: Tainan 32 past week,” the digit “32” actually represents the number of cases. However, because the word *cases* is omitted from the text, our model missed the positive sentence, which increased the false-negative rate.

**Table 3:** Error distribution of dengue case information detection.

Clause type	False positive, n (%)	False negative, n (%)	Error rate, n (%)
Single-clausal (n=456)	7 (1.5)	7 (1.5)	14 (3.1)
Multiclausal (n=509)	29 (5.7)	15 (2.9)	44 (8.6)
Corpus (n=965)	36 (3.7)	22 (2.3)	58 (6)

The goal of this research was to automatically generate a summary of the alerting articles from ProMED-mail to help researchers reduce reading effort and more quickly comprehend the main topic. Given an alerting report, our model can extract crucial sentences that express dengue case information, and these extracted sentences can be combined to form a summary. To estimate the practicality of the proposed model for epidemic monitoring, we conducted a satisfaction analysis experiment to assess the acceptability of the summaries by end users from the medical science field. The survey participants were 2 female students from the School of Public Health, Taipei Medical University; 2 male faculty members from the department of parasitology and tropical diseases, Taipei Medical University; and 1 male internal medicine physician from a Taipei Medical University–affiliated hospital; their ages ranged from 23 to 55 years. They evaluated on a 5-point Likert scale the quality of the summaries generated by our method. The summaries were randomly sampled from 25% (5/20) of the Southeast Asia dengue alerting reports from January 2019 to December 2020. We estimated the average number of words and sentences from both the original alerting reports and the generated summaries and then derived the compression rates at the word level (2.8) and sentence level (3.7). We included three questionnaire items: (1) completeness—*completeness of the generated summary content*, (2) readability—*fluent and easy to read*, and (3) helpfulness—*helps to improve analysis efficiency or reduce text reading time*.

**Figure 5:**
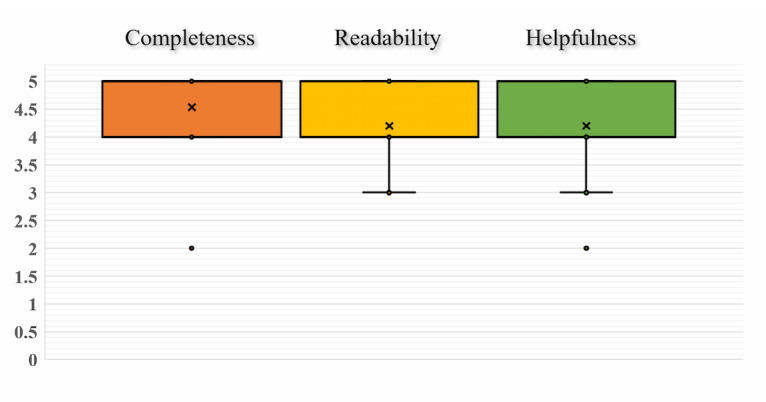
Box plot of expert assessment on a 5-point Likert scale of the quality of generated summaries.

To analyze the bias of the scoring distribution, we used a box plot to illustrate the distribution of the scores in the satisfaction survey (Figure 5). The extended range of the boxed image (including box whiskers) represents the highest to lowest distribution of the 5 scores, and the symbol *x* in the box indicates the mean value of the scores. As we can see, the item *Completeness* has the highest score, which indicates that all the epidemiologists were satisfied with the quality and accuracy of the summary text. The average scores of the remaining 2 items are also >4.2, which demonstrates that our automatic summaries are of high quality. These findings are evidence of the usefulness of this research. Nevertheless, there are still a few outliers with scores of 2 in the satisfaction questionnaire, which means that the epidemiologists were not entirely satisfied with these case summaries. On the basis of further analysis of the results, it was found that this was mainly due to incorrect results of our prediction of multiclausal sentences, which is the main type of error in the proposed model. However, multiclausal sentences typically entail rich information. Therefore, as the quality of the summary is sensitively affected by the incorrect identification of multiclausal sentences, one of our directions for future research is to improve the accuracy of multiclausal sentence prediction.

To summarize, the experiment results from the satisfaction analysis demonstrate that our summary system is helpful for experts and scholars to quickly read and effectively analyze a large number of briefings.

### Limitations and Future Directions

This study includes some limitations. The approaches developed in this study mainly focus on extracting sentence information for summarizing or, more specifically, extracting qualitative information from unstructured content. However, this approach is currently unable to acquire precise quantitative information. For instance, the number of incidence cases (newly infected) and cumulative cases cannot be reliably identified using current algorithms. Our future work will therefore focus on this issue by integrating date, location, and identification of the number of cases to retrieve important quantities of disease information. This information can be applied to more advanced spatial and temporal analyses in the future.

The second limitation is that the reporting effort and frequency in ProMED-mail are not consistent because of its volunteer-oriented design. This problem could be overcome by integrating other outbreak-reporting platforms such as the HealthMap project, which provides a visualized platform for various disease alerts [53,64]. Collecting epidemiological surveys from the scientific literature is another approach that can be used to enrich the data set.

### Conclusions

The combination of high rates of international travel and rapid environmental changes makes region-wide collaboration in monitoring emerging and re-emerging infectious diseases necessary. The current COVID-19 pandemic has also had a huge impact on the surveillance and control of other infectious diseases [65]. In addition, because ProMED-mail records and follows up undiagnosed diseases in different countries [66], this abundant disease surveillance information is unstructured and is thus not able to be efficiently used by public health workers or scientists. Our proposed deep neural network provides a good way to extract outbreak information from unstructured text, which can then be further analyzed.

In summary, our study built a prototype of an NLP algorithm to retrieve sentence summarizations from the ProMED-mail database. This approach can help medical scientists and public health workers to save more time on content summarization and analysis. Further work will continue to optimize the algorithm to extract more important quantification information.

## Abbreviations

BiLSTM: bidirectional long short-term memory
CNN: convolutional neural network
DHF: dengue hemorrhagic fever
DT: decision tree
DuBiLSTM: dual-channel bidirectional long short-term memory
LLR: log-likelihood ratio
LSTM: long short-term memory
MLP: multilayer perceptron
NLP: natural language processing
POS: parts of speech
ProMED: Program for Monitoring Emerging Diseases
ReLU: rectified linear unit
RF: random forest
SVM: support vector machine
XGB: extreme gradient boosting
